# Transabdominal preperitoneal (TAPP) *versus* open Lichtenstein hernia repair. Comparison of the systemic inflammatory response and the postoperative pain^1^

**DOI:** 10.1590/s0102-8650201900206

**Published:** 2019-02-28

**Authors:** Milton Rigoberto Fonseca Quispe, Wilson Salgado

**Affiliations:** I Fellow PhD degree, Department of Surgery, Dr. Enrique Garcés Hospital, Equador. Conception and design of the study; acquisition, analysis and interpretation of data; technical procedures; statistics analysis.; II PhD, Associate Professor, Department of Surgery and Anatomy, Clinical Hospital, Faculty of Medicine, Universidade de São Paulo (USP), Ribeirao Preto-SP, Brazil. Conception and design of the study, analysis and interpretation of data, manuscript preparation and writing, critical revision, final approval.

**Keywords:** Acute-Phase Proteins, Hernia, Inguinal, Laparoscopy, Visual Analog Scale

## Abstract

**Purpose:**

To compare open Lichtenstein repair and laparoscopic transabdominal preperitoneal (TAPP) repair to treat primary unilateral hernia, regarding systemic inflammatory response, postoperative pain, and complications.

**Methods:**

A non-randomized prospective cohort study, with the preoperative and postoperative (24 hours) collection of blood samples for C reactive protein (CRP), interleukin 6 (IL-6), leukocyte and neutrophil analysis. Visual Analog Scale (VAS) was used to quantify the level of pain, and the operative time was correlated with the inflammatory response. VAS and CRP were also obtained on the 8th postoperative day.

**Results:**

Groups were homogeneous regarding preoperative characteristics. There were no differences between groups in 24h values of CRP, IL-6, leukocytes, neutrophils or VAS. Similarly, CRP and VAS did not differ between groups on the 8th postoperative day. However, the operative time for laparoscopic hernia repair was longer than the time for the open procedure. There was a weak correlation (r coefficient 0.31) between the duration of the surgical procedure and the VAS score at the eighth day.

**Conclusions:**

There were no statistically significant differences in the inflammatory response, pain scores, or complications between groups. We conclude that there is no advantage performing a primary unilateral hernia repair by laparoscopy.

## Introduction

 More than 20 million inguinal hernia repair surgeries are performed every year worldwide. Nearly 800.000 are performed in the United States. This is a multifactorial disease affecting individuals of all ages and of both sexes. Thirty percent of patients with inguinal hernia are asymptomatic, and up to 50% are aware of their hernia. Three percent of the patients present incarceration. Indirect hernia corresponds to more than 70% of cases among adults. The recurrence after surgery ranges from 3 to 8%^1^.

 Any surgical intervention courses with inflammatory reactions of different degrees according to the length of the aggression, influencing postoperative outcomes (such as acute and chronic pain). These outcomes are determined by the degree of surgical dissection, the use of foreign material (wires, mesh, tacker), the presence of postoperative complications (seromas, hemorrhages), recurrence, and local incarceration of nerves. These complications cause an increase in hospitalization, prolonged use of anti-inflammatory drugs, increasing the hospital length of stay, with an impact on satisfaction and quality of care^2^. 

 With the surgical lesion, a cascade of neuroendocrine activation occurs minutes after the trauma that activates the sympathetic nervous system, with an immediate increase in catecholamine that causes symptoms such as high blood pressure, tachycardia, etc. At the same time, there is an increase of corticotropin stimulating the adrenal cortex, with the synthesis of cortisol^2^. This process also stimulates the production of pro- and anti-inflammatory substances which eventually end up causing a systemic inflammatory response^3^. 

 Interleukin-6 (IL-6) levels increase due to local and systemic inflammation after surgery, peaking after 4 hours and then decreasing after 48 hours. This rapid increase also produces aggressive liver protein secretion, causing activation of myeloid cells and C-reactive protein (CRP). The latter peaks between 4 and 6 hours, decreasing between 48 and 72 hours. These two markers are considered to be highly sensitive and are measurable in inflammation^4^ ^-^ ^6^. 

 There is a constant discussion among surgeons about which hernia repair technique is better: open or laparoscopic? The National Institute for Health and Care Excellence guidelines recommend open surgical approaches for the treatment of primary unilateral inguinal hernias. However, there is a trend among surgeons to perform a laparoscopic procedure. The possible advantages should be less postoperative pain and a faster recovery. But this is still a controversial matter^7^. 

 There is still a controversy about the inflammatory response produced in the open and laparoscopic hernia repair, due to the lack of prospective studies, systematic reviews and meta-analyses. It is important to evaluate the real benefits of the laparoscopic unilateral hernia repair since there is an increase in the costs.

 The objective of the present study was to compare the systemic inflammatory response (IL-6, CRP, leukocyte and neutrophil count), the postoperative pain intensity, and the rate of complications between open inguinal hernia repair (Lichtenstein) and the laparoscopic (transabdominal preperitoneal - TAPP) technique.

## Methods

 The protocol was approved by the Bioethics Committee of the Central University of Equador and the Research Ethics Committee of Hospital Dr. Enrique Garces and Hospital Dr. Gustavo Domínguez Zambrano. All patients gave written informed consent to participate in the study.

 A non-randomized prospective cohort study was performed at the two Hospitals in Equador, with patients on a day-hospital treatment regimen that met the following inclusion criteria: primary unilateral inguinal hernia (Nyhus I, II, III A and IIIB), age ranging from 18 to 70 years, ASA I or II, and body mass index (BMI) less than 30 kg/m^2^. Patients with any kind of immunosuppression or using some immunosuppressive drug, and patients with inguinal hernia demanding emergent surgery repair were excluded from the study.

 Sample size was calculated using the EPIDAT 3.1 statistical program, based on the results of the study of Vatansec *et al*.^8^. A 99% confidence level and a 95% power were set, with a required sample of 19 subjects for each arm of the study being obtained. Considering a 10% loss of cases, it was decided to include a total of 21 patients in each study group.

 Two study groups were defined: inguinal hernia repair with the open (Lichtenstein) technique and with the laparoscopic (transabdominal preperitoneal - TAPP) technique. Duration of the surgery was recorded. Lichtenstein repair was performed under spinal anesthesia with a 5 cm incision, polypropylene mesh (lightweight, 7.5 x 15 cm, BioMesh™) fixation with 2.0 polyglactin 910 separate sutures, and with no supplemental local anesthesia. TAPP required general anesthesia, with a three trocar access. The same kind of mesh was used (15 x 15 cm) but was only positioned and not fixed in the preperitoneal space. All patients received equal analgesia at induction of anesthesia and during the immediate postoperative period (Tramadol IV 100 mg and Ketorolac IV 30). They were discharged with a 5-day home prescription of oral 500 mg Paracetamol and 400 mg oral Ibuprofen every 8 hours. A search for surgical complications was performed until the 30^th^ postoperative day.

 Preoperative demographic characteristics were obtained from each patient. Peripheral 10 ml blood samples were collected during the immediate preoperative period and 24 hours after surgery in order to quantify IL-6, CRP and white blood cell counts. Samples were immediately sent to the Central Laboratory for their respective analysis. The Visual Analog Scale (VAS) was used to quantify the level of pain at the time of discharge, 24 h after surgery. VAS and CRP were also analyzed on the 8^th^ postoperative day. 

 For IL-6 analysis, the plasma obtained with lithium heparin, EDTA BI and tripotassium citrate was submitted to the electrochemiluminescence process in an Elecsys and cobas analyzer. The Sandwich technique was used, with a total duration of 18 minutes. A 30 µL sample was incubated with the anti-IL-6 biotinylated monoclonal antibody labeled with ruthenium chelate and with streptavidin-coated microparticles. These antibodies and the sample antigen form the sandwich complex; the mixture was transferred to the reading cell where the microparticles were fixed to the surface of the electrode by magnetism. The unfixed elements were eliminated with the ProCell/Procell M reagent and a defined electric current was applied, producing the chemiluminescent reaction whose light emission was measured with a photomultiplier. The results were obtained by constructing a calibration curve at two points and a master curve included in the reagent barcode. Calibration was determined by traceability and the Preci Control Multimarker was used for quality control. The result, in pg/mL, was obtained automatically, with values ranging from <1.5 pg/mL to >5000 pg / mL. Accuracy was determined with the Elecsys reagents for samples and controls according to the protocol (EP5-A2), with an average of 17.3 pg/mL in human serum, receptivity DE 1.03, CV6%, intermediate Precision DE1.46, and CV 8.5%.

 For the CRP assay, synthetic particles coated with the CRP antibody (AbPR) in goat anti CRP liquid wells, plus two microbial inhibitors and polyglycol as a buffer added to the CRP of the sample before centrifugation and complete clot formation. The samples were kept separate for 8 hours at room temperature. The Dimension system automatically performs the reagent dispensing, separation, processing, and printing of the results for a 3 µl sample, plus bichromatic type measurement and CRP type calibration. The analytical measurement range was 0.2-12 mg/dL for a mixture containing antibody at concentrations ranging from 12 to 80 mg/dL (120-800 mg/l). Sensitivity was 0.2 mg/dL and specificity per sample was greater than 10%.

 And for hematic biometry a whole blood sample was used without dilution or previous treatment with EDTA. A 100 µl sample was used for the flow cytometry process (multiparametric cellular analysis). The method was the combination of lateral, frontal and fluorescence dispersion (DNA and RNA concentration) of nucleated cells and the reagents used were Cellpack, Lisercell WNR, and Liser cell WDF. The analysis was automatically performed by the SISMEX XN1000 analyzer according to insertion of the reagent, reference value M/H4.80-10.00/mm^3^. Analytical sensitivity was <0.01. 

 Data were analyzed statistically using the SPSS software version 22 (IBM Corp. Released 2013. IBM SPSS Statistics for Windows, Version 22.0. Armonk, NY: IBM Corp.). First, variables were tested for normality using Shapiro-Wilk test Chi-squared test for categorical variables, Unpaired t-test for parametric continuous variables and Mann Whitney for non-parametric continuous variables were used. In order to evaluate correlations between variables, Spearman’s rank correlation coefficient was used. Level of significance was set at p<0.05.

## Results

 A total of 59 patients, 11 women (18.6%) and 48 men (81.4%), were included in the study, 37 submitted to Lichtenstein hernia repair (62.7%) and 22 toTAPP hernia repair (37.3%). Mean patient age (+SD) was 51.8 ± 13.02 years. Groups were similar regarding demographic characteristics and preoperative laboratory tests (Table 1) and also according to the Nyhus Classification System (Table 2).

**Table 1:** Preoperative characteristics and inflammatory response in both groups: Liechtenstein and laparoscopic transabdominal preperitoneal (TAPP) hernia repair.

	LICHTENSTEIN	TAPP	p
Age (years)	53.01 ± 13.42 (n=37)	49.77 ± 12.35 (n=22)	0.29
Male gender (%)	81.12 (n=37)	81.83 (n=22)	0.94
Leukocytes (/mm^3^)	6,033.70 ±1,566.99 (n=37)	6,932.73 ± 1,802.13 (n=22)	0.13
Neutrophils (%)	51.88 ± 8.21 (n=37)	51.61 ± 7.67 (n=22)	0.90
CRP (mg/dl)	0.40 ± 0.28 (n=33)	0.36 ± 0.35 (n=22)	0.29
IL-6 (pg/ml)	6.71 ± 2.71 (n=35)	7.71 ± 2.80 (n=22)	0.18

TAPP - transabdominal preperitoneal hernia repair; CRP- C-reactive protein; IL-6 - Interleukin 6.

**Table 2:** Nyhus intraoperative hernia classification in both groups: Liechtenstein and laparoscopic transabdominal preperitoneal (TAPP) hernia repair.

	LICHTENSTEIN	TAPP
Nyhus II	10 (27%)	6 (27%)
Nyhus III A	10 (27%)	6 (27%)
Nyhus III B	17 (46%)	10 (46%)

TAPP - transabdominal preperitoneal hernia repair.

 White blood counts, inflammation markers and VAS pain scores did not differ between groups 24 hours after surgery. CRP and VAS scores did not differ significantly between groups on the 8^th^ postoperative day (Table 3). 

**Table 3:** Postoperative inflammatory response and pain visual analog scale (VAS) in both groups: Liechtenstein and laparoscopic transabdominal preperitoneal (TAPP) hernia repair.

	LICHTENSTEIN	TAPP	p
Leukocytes 24 h (/mm^3^)	10,478.24 ± 2,176.09 (n=34)	9,951.36 ± 2,927.20 (n=22)	0.49
Neutrophils 24 h (%)	69.75 ± 11.53 (n=34)	68.49 ± 9.32 (n=22)	0.43
CRP 24 h (mg/dl)	1.68 ± 2.93 (n=33)	1.65 ± 1.99 (n=22)	0.76
CRP 8^th^ day(mg/dl)	0.47 ± 1.83 (n=33)	0.72 ± 1.83 (n=22)	0.48
IL-6 24 h (pg/ml)	19.33 ± 11.52 (n=32)	20.02 ± 9.93 (n=22)	0.98
VAS 24 h	4.11 ± 1.71 (n=37)	4.00 ± 1.41 (n=22)	0.94
VAS 8^th^ day	3.70 ± 1.59 (n=37)	3.91 ± 1.54 (n=22)	0.45

TAPP - transabdominal preperitoneal hernia repair; CRP- C-reactive protein; IL-6 - Interleukin 6; VAS - Visual analog scale.

 With regard to the duration of the surgical procedure, 58 samples were analyzed (98.30%), resulting in a mean ± SD time of 86.29 ± 28.96 minutes. The duration of surgery for the open repair group was 71.94 ± 16.48 minutes, a significantly lower value than for TAPP hernioplasty which lasted 109.77 ± 29.90 minutes (p<0.01). An experienced surgeon always participated in each operation. 

 In terms of postoperative complications, a total of 58 patients was analyzed (98.30%). Nineteen minor postoperative complications were detected (32.8%), 10 of them belonging to the open hernioplasty group, representing 27.8% of complications within that group, and 9 of them belonging to the laparoscopic group, representing 40.9%. All complications were clinically treated with an exception in the case of recurrence in the Lichtenstein group that needed reoperation, and that was diagnosed as a femoral hernia. There was no statistically significant association between the surgical procedure and the incidence of postoperative complications (p = 0.30) (Table 4).

**Table 4:** Complications in both groups: Liechtenstein and laparoscopic transabdominal preperitoneal (TAPP) hernia repair.

	LICHTENSTEIN	TAPP
Seroma	7 (19.47%)	4 (18.18%)
Hematoma	2 (5.55%)	3 (13.63%)
Orchitis	0 (0%)	1 (4.54%)
Recurrence	1 (2.7%)	0 (0%)

TAPP - transabdominal preperitoneal hernia repair.

 Patients with complications had higher CRP values on the 8^th^ postoperative than patients with no complications (1.10 mg/dl *vs*. 0.29 mg/dl; p = 0.03). Similarly, on the same postoperative day, VAS values were higher for the group with complications (44.32 *vs.* 22.28; p < 0.01). There were no differences between groups in the values of IL-6, leukocytes, neutrophils, CRP and VAS 24 hours after surgery. There was a correlation (r coefficient 0.31, p=0.01) between the duration of the surgical procedure and the VAS score at the eighth day. 

## Discussion

 We found few studies comparing the systemic inflammatory response between Lichtenstein hernioplasty and TAPP laparoscopic hernioplasty, with significant variability of results and conclusions. Nor is there a standardization of the inflammatory markers that could be used to quantify the postoperative inflammatory response, further increasing the variability of findings^9^ ^-^ ^12^.

 Some studies comparing the inflammatory response between TAPP and Liechtenstein hernia repair have reported that laparoscopic surgeries caused a lower systemic cytokine response, a result attributed to the lesser extent of surgical trauma^9^. Similarly, studies by Suter *et al.* ^10^, Bender *et al.* ^11^, and Bugada *et al.* ^13^ demonstrated a lower inflammatory response with TAPP compared to other open techniques of hernia repair such as Nyhus, Lichtenstein, Stoppa, and Bassini. However, our study found no statistically significant differences between groups, in agreement with the results obtained by Hill *et al.* ^12^, who reported that the inflammatory response is not modified when performing laparoscopic inguinal hernia repair. Equally, Schrenk *et al.* ^14^ found the same results, but with less pain with the laparoscopic technique. 

 Akhtar *et al.* ^2^ and Takahara *et al.* ^15^ reported that CRP concentration increased significantly on the first postoperative day in groups of open and laparoscopic hernia repair, and that this increase was higher in the open group. Schrenk *et al.* ^14^ found a significant increase in CRP and fibrinogen concentrations on the first and second postoperative days compared to reference values, but with no differences between groups. It was reported that an increase in CRP is proportional to the severity of surgical trauma and indicates the magnitude of tissue destruction^16^. These results show that tissue damage is substantial in the open hernia repair, but also in the laparoscopic technique. In this respect, Schwab *et al.* ^17^ pointed out that, unlike other types of laparoscopic surgery, hernioplasty performed by this technique should not be considered a minimally invasive surgery and could be considered, perhaps, a little less traumatic than conventional approaches. It has also been reported that repair with the Shouldice technique produces a lower inflammatory response, mainly if it is carried out under local anesthesia.

 The controversy about the inflammatory response produced in the open and laparoscopic hernia repair persists due to lack of prospective studies, systematic reviews and meta-analyses. In the current study, one of the justifications of the values of TAPP surgery being equivalent to those of the open technique was probably the longer surgical time of the technique.

 According to several authors, laparoscopic surgical treatment of bilateral inguinal hernia apparently shows a lower rate of postoperative pain, reduction of the time of disability, reduction of hospital stay, and low postoperative complications, therefore allowing a better quality of life^18^. With regard to postoperative pain and complications, in the present study of unilateral hernias, we found no difference between groups.

 This study did not compare the real costs of the two types of surgery. However, in view of the longer duration and the fixed costs of laparoscopy, we could observe that this technique was more expensive. In addition, considering that there were no statistically significant differences in inflammatory response, complications or pain scores between groups, we may conclude that, in our service, there is no advantage to perform a primary unilateral hernia repair by laparoscopy. A limitation of the study that should be pointed out was the lack of patient randomization, although the minimal sample needed for statistical representativeness was calculated and the cohorts studied were preoperatively comparable.

## Conclusions

 There were no statistically significant differences in the inflammatory response, pain scores, or complications between groups. We conclude that there is no advantage performing a primary unilateral hernia repair by laparoscopy**.**
